# Nutritional and lifestyle risk behaviors and their association with mental health and violence among Pakistani adolescents: results from the National Survey of 4583 individuals

**DOI:** 10.1186/s12889-015-1762-x

**Published:** 2015-04-28

**Authors:** Saadiyah Rao, Nadia Shah, Nida Jawed, Sumera Inam, Kashif Shafique

**Affiliations:** School of Public Health, Dow University of Health Sciences, Karachi, 74200 Pakistan; Institute of Health and Wellbeing, Public Health, University of Glasgow, 1-Lilybank Gardens, Glasgow, G12 8RZ UK

**Keywords:** Unhealthy behaviors, Adolescents, Mental health, Physical fight

## Abstract

**Background:**

*Unhealthy behaviors* are associated with mental health problems and violence in adolescents, yet their combined association has been understudied. Using the Global School Health Survey, this study examined the association between combined *unhealthy behaviors* (including fast food, soft drink, smoking, other tobacco products and physical inactivity) and anxiety, suicidal ideation and involvement in physical fight among Pakistani adolescents.

**Methods:**

Data were obtained from the Global School Health Survey conducted in Pakistan (2009). The study population consisted of school going adolescents aged 13 to 15 years. Association of combined *unhealthy behaviors with* anxiety, suicidal ideation and involvement in physical fight were studied through secondary analysis. We used univariate and multivariate logistic regression analysis by complex sample method, accounting for cluster sampling technique used for data collection.

**Results:**

Of the total 4583 students, weighted percentage and unweighted count for *one, two, three* and *four* or *more unhealthy behaviors was* 39.4% (n = 1770), 22.1% (n = 963), 5.9% (n = 274) and 1.2% (n = 62) respectively. The weighted prevalence for anxiety, suicidal ideation and involvement in physical fight were 8.4%, 7.3% and 37.4% respectively. The results of multivariate logistic regression analysis after adjustment showed that students who had four or more *unhealthy behaviors* had higher odds of; being anxious (OR 2.45, 95%CI 1.31-4.59, *p* value 0.004), suicide ideation (OR 4.56, 95%CI 2.58-8.07, *p* value <0.001) and being involved in physical fight (OR 3.15, 95% CI 1.63–6.08, *p* value <0.001) as compared to those who had not adopted any *unhealthy behaviors*.

**Conclusions:**

This study suggests that the co-occurrence of unhealthy behaviors is associated with anxiety, suicidal ideation and physical fight among adolescents. These findings should be considered when developing interventions to combat detrimental outcomes of *unhealthy behaviors* during adolescence*.*

## Introduction

Evidence suggests that the disparities in morbidity and mortality which exist among different population groups cannot be fully explained by biomedical factors. However, these disparities can be further understood through the study of lifestyle patterns which are shaped by various social and behavioral components [1]. Lifestyle behaviors usually originate during adolescence; a period of growth and development where new habits and behaviors are established through experimentation. These acquired behaviors might persist throughout life and could contribute towards the development of adverse health effects in adulthood [2].

In Pakistan (where adolescents comprise of about 22% of population) [3] *unhealthy behaviors* like fast food consumption, smoking and physical inactivity have increased dramatically due to rapid urbanization and western influence. Approximately 29.5% adolescents consume fast food; 9.6% have sedentary lifestyle; [4] 9.8% use tobacco products; and 6.3% smoke cigarettes; [5] and 29.4% consume soft drinks regularly [6]. In parallel, there is also a growing trend of physical fight in adolescents along with rise in mental health problems [7,8]. Associations between certain *unhealthy behaviors* such as dietary consumption of fast food & soft drinks, smoking, use of tobacco products and physical inactivity and mental health problems like depression, anxiety and suicidal ideation have been identified in literature [9-13]. Likewise, smoking in particular has been repeatedly linked with aggression and interpersonal violence among adolescents [14,15]. While some protective factors like parental involvement and having close friends have been associated with a decreased risk of mental health problems among adolescents [16-18].

The impact of specific *unhealthy behavior* on different health outcomes has been reported by various studies. However, studying single behavior might be an overly simplistic approach, since evidence has shown that *unhealthy behaviors* do not occur in isolation rather they co-occur as lifestyle patterns [19]. This co-occurrence of *unhealthy behaviors* appears to generate synergistic effects, rather than the aggregated effects of the individual behavior. Although, numerous studies have focused on identification of patterns or clusters of *unhealthy behaviors* in population, however more evidence is needed to examine their association with various health outcomes [20]. Thus, addressing multiple *unhealthy behaviors* collectively has been advocated to increase efficacy and decrease cost of preventive interventions curtailing the adverse health outcomes.

Keeping in view the limited contextual evidence regarding effects of *unhealthy behaviors* on mental health problems and physical fight among adolescents, this study aimed to investigate the association of combined unhealthy lifestyle behaviors including fast food and soft drink intake; smoking and other tobacco products use; and physical inactivity with anxiety, suicidal ideation and involvement in physical fight among school going adolescents in Pakistan.

## Methods

### Study setting and sample

Pakistan is a lower middle income country in South Asian region, with the world’s sixth largest population (approximately 180 million). Current population structure of Pakistan is bolstered by adolescents with more than one third of the population under 15 years of age [21].

The Global School Health Survey (GSHS), a surveillance project aimed to assist countries to measure the behavioral risk and protective factors among school going adolescents. This study involved the secondary analysis of GSHS 2009 data collected in Pakistan by the Ministry of Health in collaboration with the World Health Organization (WHO) and Centers of Disease Control and Prevention (CDC) [22].

A nationally representative sample of students in grades 8 to 10 was obtained through two-stage cluster sampling. In the first stage, the schools were selected based on probability proportional to their enrollment size. In the second stage, the classes were randomly selected and all students in selected classes were eligible to participate in the survey.

A self-completed questionnaire was used. A total of 5192 students participated in the survey, giving a response rate of 76%. Informed written consent was obtained from the parents/guardians of respondents in advance. GSHS was reviewed and approved by Local Ethical Review Committee (LERC) of each participating country [23].

### Measures

GSHS covers the global leading causes of morbidity and mortality among children and adults. The 11 core questionnaire modules included questions on alcohol use, dietary behaviors, drug use, hygiene, mental health, physical activity, protective factors, respondent demographics, sexual behaviors, tobacco use and violence [22]. However, the Pakistan GSHS assessed only dietary behaviors; hygiene; mental health; physical activity; protective factors; tobacco use; and violence. All details including questionnaire, codebook and data for the survey used is available at http://www.cdc.gov/gshs/countries/eastmediter/pakistan.htm. All the measures in our study were dichotomized based on definitions used in previous studies [4,14,24].

### Socio-demographic measures

Gender and age of the participants were included as recorded in the survey. While, socioeconomic status (SES) was measured by the standard variable, *“During the past 30 days, how often did you go hungry because there was not enough food in your home?”* [25] (Options never to rarely were recoded as ‘average’, and options, sometimes to always as ‘below average’).

### Physical measures

The body mass index (BMI) was calculated as weight (Kgs)/height (m^2^). It was categorized according to WHO standard BMI percentiles for children which classifies normal weight as 5th to 84th percentile and overweight as 85th and above.

### Unhealthy behavior measures

We combined *unhealthy behaviors* based on dietary consumption of carbonated soft drinks and fast food, tobacco use (smoked and other tobacco products) and physical inactivity, measured through five items including:

- *“During the past 30 days how many times per day did you usually drink carbonated soft drinks, such as Pepsi, Coca cola, 7-up, Sprite, Fanta and Dew?”*[25] (Recoded: I did not drink and <1 times/day as ‘no’ and 1 to >5 times/ day as ‘yes’).
- *“During the past 7 days on how many days did you eat food from a fast food restaurant such as McDonalds, KFC, Pizza Hut, Subway, AFC, or Al- baik?”* [25] *(*Recoded: 0 days as ‘no’ and 1 to 7 days as ‘yes’).
- *“During the past 30 days, on how many days did you smoke cigarettes?”* [25] (Recoded: 0 as ‘no’ and 1 to 30 days as ‘yes’).
- *“During the past 30 days, on how many days did you use any other form of tobacco, such as hukka, bidi, niswar, shisha, or mainpuri (a pasty material made of crushed tobacco, beetle nut, and catechu)?”* [25] (Recoded: 0 days ‘no’ 1 to 30 days as ‘yes’).
- *“How much time do you spend during a typical day sitting and watching television, playing computer games, talking with friends, or doing other sitting activities such as playing carom board, luddo, or cards?”* [25] (Recoded: <1 hour as ‘no’ and 1 to ≥8 hours as ‘yes’).

Respondents scored one point for each item and were classified as having *one*, *two*, *three* and *four* or *more unhealthy behavior*s, whereas zero point was categorized as *‘none’.*

### Protective factor measures

Following three protective factors were also included:

- Close friends: *“How many close friends do you have?”* [25] (Recoded: 0 as ‘no’ and 1 to ≥3 as ‘yes’).
- Parental check: *“During the past 30 days, how often did your parents or guardians really know what you were doing with your free time?”* [25] (Recoded: never to rarely as ‘no’ and sometimes to always as ‘yes’).
- Parental understanding: *“During the past 30 days, how often did your parents or guardians understand your problems and worries?”* [25] (Recoded: never to rarely as ‘no’ and sometimes to always as ‘yes’).

### Outcome measures

We considered mental distress (anxiety and suicidal ideation) and physical fight as the three separate dependent variables.

- Anxiety was assessed from the item “*During the past 12 months, how often have you been so worried about something that you could not sleep at night?”* [25] (Recoded: never to sometime as ‘no’ and often to always as ‘yes’).
- Suicidal Ideation: “During the past 12 months, did you make a plan about how you would attempt suicide?” [25] (Recoded: 0 as ‘no’ and 1 as ‘yes’).
- Physical fight: *“During the past 12 months, how many times were you in a physical fight?”*[25] (Recoded: 0 times as ‘no’ and 1 to ≥12 times as ‘yes’).

### Statistical analysis

We obtained the dataset from the CDC website and analyzed it through SAS 9.1.3 computer package (Statistical Analysis System, RTI, Cary, North Carolina, USA). To assure the true representation of population, ‘complex samples’ method was used for weighted analysis accounting for cluster sampling technique used in GSHS. Statistical analysis included chi-square, and binary logistic regression with significance level of 0.05 to examine the associations between unhealthy behaviors and the explanatory variables. Multivariate analysis for each outcome variable was separately done after controlling for the rest of the predictor variables, which included age, gender, BMI, SES, parental understanding, parental check and close friends. We constructed our final dataset using information from 4583 participants out of 5192, since 609 participants were excluded due to missing data. However, preliminary analysis did not show any difference in baseline characteristics of the missing group. We thereafter reported our results in the form of weighted percentages, unweighted counts and odds ratios.

## Results

In this study, the median age of adolescents was found to be 14 years with 39.1% (n = 1150) females. Of the total respondents, 35.9% (n = 1490) consumed soft drink, 20.7% (n = 931) ate fast food, 6.1% (n = 331) smoked cigarette, 6.0% (n = 319) used other tobacco products and 37.7% (n = 1707) were physical inactive. While 39.4% (n = 1770), 22.1% (n = 963), 5.9% (n = 274) and 1.2% (n = 62) reported one, two, three and four or more *unhealthy behaviors* respectively. Whereas, 31.3% (n = 1514) did not adopt any *unhealthy behaviors.* The weighted prevalence for anxiety, suicidal ideation and involvement in physical fight was 8.4%, 7.3% and 37.4% respectively.

### Anxiety

Table 1 depicts that *unhealthy behavior* (*p* value <0.001), age (*p* value 0.009) and SES (*p* value <0.001) and close friends (*p* value 0.008) were statistically significantly associated with anxiety. Table 2 represents univariate analysis showing that the students with ‘two *unhealthy behaviors’,* ‘three *unhealthy behaviors’* and ‘four or more *unhealthy behaviors’* were more likely to be anxious as compared to students reporting none of the *unhealthy behaviors.* Multivariate logistic regression analysis showed (represented in Table 3) slight difference in odds ratio for all the factors that were significant in the univariate analysis. Adjusted odds ratio for ‘three *unhealthy behaviors’ was (*OR 1.8195% CI1.22-2.70, *p* value 0.003), and ‘four *unhealthy behaviors’* was (OR 2.4595% CI 1.31-4.59, *p* value <0.004) as compared to no *unhealthy behavior*.

**Table 1:** **Baseline characteristics among adolescents in Pakistan**

**Characteristics**		**Anxiousness**			**Suicidal ideation**			**Physical fight**		
		No%(n)**	Yes %(n)**	**p-value**	No%(n)**	Yes %(n)**	**p-value**	No%(n)**	Yes %(n)**	**p-value**
*Unhealthy behavior										
	No	31.6(1399)	28.2(115)	<0.001	31.9(1427)	24.9(87)	<0.001	32.6(945)	29.2(569)	<0.001
	One	40.1(1652)	31.7(118)		39.9(1654)	33.2(116)		39.5(1046)	39.2(724)	
	Two	21.6(859)	28.4(104)		21.9(882)	25.2(81)		22.5(568)	21.5(395)	
	Three	5.6(235)	9.4(39)		5.3(236)	12.9(38)		4.7(129)	7.8(145)	
	Four or more	1.1(52)	2.4(10)		1.0(49)	3.9(13)		0.6(20)	2.2(42)	
**Gender**										
	Male	61.2(3161)	57.5(272)	0.472	60.8(3184)	61.8(249)	0.779	51.8(1831)	76.1(1602)	<0.001
	Female	38.8(1036)	42.5(114)		39.2(1064)	38.2(86)		48.2(877)	23.9(273)	
**Age (years)**										
	≤14	61.6(2517)	53.8(208)	0.009	61.2(2535)	57.5(190)	0.223	62.0(1606)	59.3(1119)	0.332
	≥15	38.4(1680)	46.2(178)		38.8(1713)	42.5(145)		38.0(1102)	40.7(756)	
**Body Mass Index**										
	Normal weight	84.0(3568)	82.0(320)	0.289	84.0(3610)	82.0(278)	0.412	83.6(2283)	84.2(1605)	0.619
	Over weight	16.0(629)	18.0(66)		16.0(638)	18.0(57)		16.4(425)	15.8(270)	
**Socio Economic Status (SES)**										
	Average	82.5(3443)	67.3(259)	<0.001	81.6(3444)	77.1(258)	0.079	83.2(2228)	78.0(1474)	0.022
	Below Average	17.5(754)	32.7(127)		18.4(804)	22.9(77)		16.8(480)	22.0(401)	
**Close friends**										
	No	7.3(288)	11.2(45)	0.008	7.2(288)	13.0(45)	0.004	8.1(209)	7.0(124)	0.141
	Yes	92.7(3909)	88.8(341)		92.8(3960)	87.0(290)		91.9(2499)	93.0(1751)	
**Parental check**										
	No	49.6(2107)	49.4(191)	0.951	49.3(2113)	53.5(185)	0.254	48.4(1329)	51.5(969)	0.173
	Yes	50.4(2090)	50.6(195)		50.7(2135)	46.5(150)		51.6(1379)	48.5(906)	
**Parental understanding**										
	No	44.5(1933)	49.7(194)	0.222	44.1(1934)	55.2(193)	<0.001	41.7(1174)	50.2(953)	<0.001
	Yes	55.5(2264)	50.3(192)		55.9(2314)	44.8(142)		58.3(1534)	49.8(922)	

*Unhealthy behavior: fast food, soft drink, smoking, other tobacco products and sedentary behavior.

**weighted % and unweighted n.

N = 4583.

**Table 2:** **Univariate analysis of anxiousness, suicidal ideation and physical fight with risk behaviors**

		**Anxiousness (Unadjusted)**		**Suicidal ideation (Unadjusted)**		**Physical fight (Unadjusted)**	
**Characteristics**		**OR (95% CI)**	**p-value**	**OR (95% CI)**	**p-value**	**OR (95% CI)**	**p-value**
**Unhealthy behavior**							
	No	1		1		1	
	One	0.88(0.62-1.26)	0.506	1.06(0.80-1.40)	0.656	1.11(0.92-1.33)	0.260
	Two	1.47(1.02-2.13)	0.038	1.47(1.04-2.07)	0.026	1.06(0.78-1.45)	0.691
	Three	1.89(1.33-2.68)	<0.001	3.07(1.66-5.71)	<0.001	1.84(1.29-2.63)	<0.001
	Four or more	2.41(1.33-4.34)	<0.001	4.88(2.84-8.39)	<0.001	3.89(2.08-7.26)	<0.001
**Gender**							
	Male	1		1		1	
	Female	1.16(0.76-1.77)	0.468	0.95(0.70-1.29)	0.782	0.33(0.23-0.48)	<0.001
**Age (years)**							
	≤14	1		1		1	
	>15	1.37(1.07-1.77)	0.012	1.16(0.91-1.48)	0.222	1.12(0.88-1.42)	0.350
**Body Mass Index**							
	Normal weight	1		1		1	
	Over weight	1.15(0.88-1.49)	0.292	1.14(0.95-1.38)	0.409	0.95(0.77-1.16)	0.629
**Socio Economic Status**							
	Average	1		1		1	
	Below Average	2.29(1.61-3.26)	<0.001	1.31(0.96-1.79)	0.087	1.39(1.03-1.87)	0.028
**Close friends**							
	No	1		1		1	
	Yes	0.62(0.43-0.89)	0.009	0.52(0.33-0.82)	0.005	1.17(0.93-1.47)	0.174
**Parental check**							
	No	1		1		1	
	Yes	1.00(0.76-1.33)	0.951	0.84(0.63-1.13)	0.259	0.88(0.73-1.06)	0.190
**Parental understanding**							
	No	1		1		1	
	Yes	0.81(0.58-1.13)	0.215	0.64(0.48-0.84)	0.001	0.70(0.60-0.83)	<0.001

*Cut off 0.25.

**Table 3:** **Multivariate analysis of anxiousness, suicidal ideation and physical fight with risk behaviors**

**Characteristics**		Anxiety (adjusted)*		Suicidal ideation (adjusted)**		Physical fight (adjusted)***	
		**OR (95% CI)**	**p-value**	**OR (95% CI)**	**p-value**	**OR (95% CI)**	**p-value**
**Unhealthy behavior**							
	No	1		1		1	
	One	0.87(0.60-1.25)	0.452	1.07(0.82-1.40)	0.596	1.24(1.03-1.48)	0.018
	Two	1.42(0.99-2.05)	0.054	1.52(1.08-2.13)	0.014	1.23(0.95-1.61)	0.111
	Three	1.81(1.22-2.70)	0.003	3.03(1.57-5.84)	<0.001	1.92(1.43-2.57)	<0.001
	Four or more	2.45(1.31-4.59)	0.004	4.56(2.58-8.07)	<0.001	3.15(1.63-6.08)	<0.001
**Gender**							
	Male	1				1	
	Female	1.26(0.90-1.77)	0.168	n/a		0.34(0.24-0.51)	<0.001
**Age (years)**							
	≤14	1		1			
	>15	1.39(1.10-1.77)	0.005	1.20(0.92-1.55)	0.164	n/a	
**Socio economic status**							
	Average	1		1		1	
	Below Average	2.21(1.58-3.10)	<0.001	1.22(0.90-1.65)	0.198	1.32(1.00-1.74)	0.047
**Close friends**							
	No	1		1		1	
	Yes	0.64(0.44-0.93)	0.021	0.52(0.32-0.83)	0.006	1.11(0.88-1.40)	0.355
**Parental check**							
	No	n/a		n/a		1	
	Yes					1.02(0.87-1.20)	0.785
**Parental understanding**							
	No	1		1		1	
	Yes	0.82(0.59-1.13)	0.235	0.67(0.50-0.90)	0.008	0.81(0.69-0.95)	0.011

*Adjusted for gender, age, SES, close friends and Parental understanding.

**Adjusted for age, SES, and Close friends and Parental Understanding.

***Adjusted for gender, SES, Close Friends, Parental understanding and Parental check.

### Suicidal ideation

*Unhealthy behavior* (*p* value <0.001), having close friends (*p* value 0.004) and parental understanding (*p* value < 0.001) were statistically significantly associated with suicidal ideation as represented in Table 1. Univariate analysis shown in Table 2, indicated that students with ‘two *unhealthy behaviors’*, ‘three *unhealthy behaviors’* and ‘four or more *unhealthy behaviors’* showed statistically significantly higher odds for suicidal ideation. Multivariate analysis (Table 3) showed that the students with ‘two *unhealthy behaviors’* (OR 1.52, 95% CI 1.08-2.13, *p* value 0.014) and ‘three *unhealthy behaviors’* (OR 3.03, 95% CI 1.57–5.84, *p* value <0.001) and ‘four or more *unhealthy behaviors’* (OR 4.56, 95% CI 2.58–8.07, *p* value <0.001) were more likely to plan suicide as compared to students having no *unhealthy behavior*. In contrast, students having close friends (OR 0.52, 95% CI 0.32–0.83, *p* value 0.006) and parental understanding (OR 0.67, 95% CI 0.50–0.90, *p* value 0.008) were less likely to plan suicide.

### Physical fight

Table 1 indicates that *unhealthy behavior* (*p* value <0.001), gender (*p* value <0.001), SES (*p* value 0.022) and parental understanding (*p* value <0.001) were statistically significantly associated with physical fight. Table 2 shows that students with ‘three *unhealthy behaviors’* and ‘four or more *unhealthy’* had higher odds of being involved in a physical fight. The results of multivariate logistic regression analysis (Table 3) showed that students who had ‘three *unhealthy behaviors’* (OR 1.92, 95% CI 1.43-2.57, *p* value <0.001) and ‘four or more *unhealthy behaviors’* (OR 3.15, 95% CI 1.63-6.08, *p* value <0.001) as compared to those who had not adopted any *unhealthy behavior* were more likely to be involved in physical fight. Moreover, students belonging to below average SES (OR 1.32, 95% CI 1.00-1.74, *p* value 0.047) were more likely to be involved in physical fight. Whereas, females (OR 0.34, 95% CI 0.24–0.51, *p* value <0.001) and those who had parental understanding (OR 0.81, 95% CI 0.69–0.95, *p* value 0.011) as compared to their counterparts were less likely to be involved in a physical fight (Table 3).

## Discussion

Our school based adolescent study, reveals a number of important findings regarding the associations of combined *unhealthy behaviors* with mental health problems and involvement in physical fight. Adolescents who had four or more unhealthy behaviors had more than twice the risk of mental health problems and were three times more likely to be involved in physical fight. Students who had close friends, and better child–parent relationship were less likely to be associated with mental health problems and were at a lower risk of being involved in physical fight.

Furthermore we estimated that the prevalence of being anxious, having suicide ideation, and reporting physical fight were 8.4%, 7.3%, and 37.4% respectively. It was also found that the likelihood of these problems increased with the increase in number of the unhealthy behaviors exhibited. The reported prevalence of being anxious and involvement in physical fight were found to be lower than other South Asian countries, like India [26] and Sri Lanka [27]. While the prevalence of suicidal ideation is comparable to China [18]. Though, due to methodological differences cross country comparisons must be carefully interpreted. Among the total adolescents, more than one third reported physical inactivity and soft drink consumption, about one fifth reported fast food intake, whereas 6% reported smoking. These findings highlight the need of public health measures that could address the rise in adaptation of unhealthy behaviors among adolescents.

Involvement in single *unhealthy behavior* might increase the possibility of involvement in other risk behaviors and when these risk behaviors combine they augment the possibility of mental health problems [19]. In this regard, our findings are also important; demonstrating that combined effect of multiple unhealthy behaviors appears to have a stronger association with suicidal ideation and anxiety. Moreover, the likelihood of mental health problems increases with the increase in number of unhealthy behaviors providing strong rationale to intervene on combined unhealthy lifestyle behaviors at the population level. A study conducted on French adult population showed that depression was associated with health risk-related behavior clusters which were based on poor diet, physical inactivity and substance abuse [9]. Similarly, an Irish study reported that adults with multiple risk factor clusters based on physical activity, smoking, diet and alcohol drinking had highest psychological distress [19]. Another study conducted in New Zealand reported that the adolescents who were smokers at early age were at a greater risk of suicidal ideation later in life [13]. Our finding supports the contention that for mental health problems, *unhealthy behaviors* carry their own risk apart from risk imposed by socioeconomic and gender differences.

The results indicate that violence-related behaviors are frequent among students and that they are positively associated with certain unhealthy behaviors. The risk of being involved in a physical fight was thrice among students who had four or more *unhealthy behaviors*. Similar findings have been documented by other studies conducted in adolescent population. The United States Youth Risk Behavior Survey reported that students who were associated with risky behaviors such as smoking, alcohol and drugs were more likely to be involved in physical fight [24]. Moreover, a Namibian study based on GSHS showed that being engaged in physical fight was associated with cigarette smoking, alcohol and illicit drug use [14]. However, comparison between various countries is difficult to conclude due to dissimilarities in *unhealthy behavior* patterns and measures.

In addition to *unhealthy behaviors*, we found that below average SES was associated with anxiety problems. These findings are consistent with other studies [16,26,27]. In spite of the fact that the low SES independently results in poor health outcomes, the risk behaviors more commonly prevail among deprived sections of society, where shared socio cultural determinants give rise to accumulation of *unhealthy behaviors,* [28] which makes the situation even more worst. We also found that female gender was associated with being anxious; strengthening the consistent evidence that females are more prone to psychological problems as compared to males [27]. Furthermore, we found that older adolescents were more likely to be worried than their younger counterparts. A comparable age related increase in the odds of being worried was observed among Indian and Sri Lankan adolescents [16,27]. In Pakistan, this trend continues with age leading to increased burden of anxiety in adulthood [7]. On contrary, adolescents who had close friend’s support were less likely to feel anxious or plan suicide; moreover parental understanding showed protective effect against suicidal ideation. Evidence has shown that having close friends protect against mental health problems since adolescents choose friends over parents for their company and emotional support [17].

Notably physical fight differed in genders, being less likely among females; this finding is consistent with international literature and reinforces the theory that male gender is more inclined towards interpersonal violence [29]. Our finding also supports that adolescents whose parents understand their problems and worries are less likely to involve in physical fight [14].

We lack considerable evidence on clustering of *unhealthy behaviors* in Pakistani adolescents therefore we not only need to focus on identification of unhealthy clusters but also have to adopt robust evaluation methods that could be used to understand the association of *unhealthy behavior* clusters with different health outcomes. This approach would provide more insight to this phenomenon and an opportunity for comparison with other populations. Also further research regarding local socio cultural settings needs to be done for examining the association between mental health problems and SES in context to *unhealthy behaviors.*

Our findings have important implication for public health preventive initiatives in adolescents because adolescence is the most crucial age to intervene for any long term behavioral modifications. In order to capture this targeted group, educational system could act as an interventional platform not only to combat detrimental outcomes of *unhealthy behaviors* in adolescents but could also provide long term beneficial effects in adulthood.

### Strengths and limitations

The strengths of this study include nationally representative sample taken through standardized questionnaire. It is the first large population based study conducted in Pakistan that has examined significant positive (harmful) associations of combined *unhealthy behaviors* with mental health problems and physical fight among adolescents. On the other hand present study suffers from several limitations. Firstly, data were collected from adolescents enrolled in schools (the secondary education enrollment rate is 35% in Pakistan [21]) therefore results could not be generalized on youngsters not attending school that remains a major constraint of our study. Secondly, as the questionnaire was self-reported, it is possible that some respondents might have misreported the questions asked. However, anonymity has minimized this possibility, since anonymous self-administration provides fairness and guarantees confidentiality therefore the respondents may choose to comfortably report the truth. Thirdly, the current measure of cigarette smoking used does not distinguish between regular or experimental smokers. Such misclassification might have overestimated the true prevalence of substance use among adolescents. Fourthly, it might also be possible that adolescents who are already depressed and anxious might be more likely to adopt poor dietary habits, physically inactive lifestyle, involved in smoking and other tobacco products use. Still it is not fully understood whether the *unhealthy behaviors* lead to mental health problems and involvement in physical fight or vice versa. Since this was a cross-sectional study we were unable to establish the direction of causality. In Pakistan, social taboos constrain the open discussion on risky sexual behavior among adolescents. It has also been reported that risky sexual behavior and illicit drug use are more common among street children [30,31]. Therefore, we were not able to include risky sexual behavior and drug abuse in our *unhealthy behaviors* as no data were collected for these aspects.

## Conclusions

In conclusion, combined *unhealthy behaviors* were significantly associated with being anxious, having suicidal ideation and being involved in physical fight among Pakistani adolescents. Our findings highlight the requirement to consider beyond the conventional approach of assessing independent associations of individual *unhealthy behavior* with mental health problems and physical fight. Further evidence is required to examine how different *unhealthy behaviors* cluster, interact and impact psychosocial health among adolescents.
